# Clinical Impact of Lesion Complexity on 2-Year Outcomes After Zotarolimus-Eluting Stents Implantation

**DOI:** 10.1016/j.jacasi.2021.08.006

**Published:** 2021-10-26

**Authors:** Jung-Ho Park, Cheol Hyun Lee, Yun-Kyeong Cho, Hyuck-Jun Yoon, Chang-Wook Nam, Jong Seon Park, Kee-Sik Kim, Hun Sik Park, Bong-Ryeol Lee, Eun-Seok Shin, Jang-Ho Bae, Young Dae Kim, Seung-Ho Hur

**Affiliations:** a Division of Cardiology, Department of Internal Medicine, Keimyung University Dongsan Medical Center, Daegu, South Korea; b Division of Cardiology, Department of Internal Medicine, Yeungnam University Medical Center, Daegu, South Korea; c Division of Cardiology, Department of Internal Medicine, Daegu Catholic University Medical Center, Daegu, South Korea; d Division of Cardiology, Department of Internal Medicine, Kyungpook National University Hospital, Daegu, South Korea; e Division of Cardiology, Department of Internal Medicine, Daegu Fatima Hospital, Daegu, South Korea; f Division of Cardiology, Department of Internal Medicine, Ulsan University Hospital, Seoul, South Korea; g Division of Cardiology, Department of Internal Medicine, Konyang University Hospital, Nonsan, South Korea; h Division of Cardiology, Department of Internal Medicine, Dong-A University College of Medicine, Busan, South Korea

**Keywords:** complex percutaneous coronary artery intervention, coronary artery disease, drug-eluting stents, CKD, chronic kidney disease, DES, drug-eluting stent(s), IVUS, intravascular ultrasound, MACE, major adverse cardiac events, MI, myocardial infarction, OCT, optical coherence tomography, PCI, percutaneous coronary intervention, TLF, target lesion failure, TLR, target lesion revascularization, ZES, zotarolimus-eluting stent(s)

## Abstract

**Background:**

The clinical efficacy and safety of second-generation drug-eluting stents in complex percutaneous coronary interventions (PCIs) are not well established.

**Objectives:**

The clinical influence of the lesion complexity after PCI with zotarolimus-eluting stents (ZES) was evaluated.

**Methods:**

From a prospective multicenter observational study, a total of 926 patients that underwent successful PCIs with ZES were included. Complex PCIs were defined as patients with ≥3 lesions treated, 3 vessels treated, severe calcified lesions, bifurcated lesions with 2 stents implanted, left main disease, chronic total occlusion lesions, and/or diffuse long (total stent length ≥60 mm) lesions and were compared to the noncomplex group. The primary outcome was incidence of target lesion failures at 2 years, defined as a composite of cardiac death, target lesion-myocardial infarctions, and target lesion revascularization.

**Results:**

The patients were divided into complex PCI (n = 249) and noncomplex (n = 677) groups. In the complex PCI group, the 2-year risk of a target lesion failure was not significantly higher than in the noncomplex PCI group (4.8% vs 3.7%; adjusted hazard ratio: 1.373; 95% confidence interval: 0.689–2.738; *P* = 0.367). The same trend was observed for all composites of the clinical outcomes. Older age and advanced chronic kidney disease were independent predictors for the primary outcome.

**Conclusions:**

Up to 2 years after a ZES implantation, the clinical outcomes did not differ according to lesion complexity.

In general, a relatively higher restenosis rate and poorer prognosis were reported in patients with complex coronary artery disease in the drug-eluting stent (DES) era, and the U.S. Food and Drug Administration on-label indication is limited to low-risk patients with stable coronary artery disease and simple single lesions (1). With the remarkable development of DESs and procedural techniques in patients with coronary artery disease, there have been significant improvements in the clinical outcomes after percutaneous coronary intervention (PCI) even in cases suitable for coronary artery bypass surgery, such as left main or 3-vessel disease (2). Resolute zotarolimus-eluting stents (ZES) (Medtronic), have been developed to improve the clinical safety and efficacy by their enhanced flexibility and deliverability and might be expected to improve the clinical outcomes even in the high-risk group (3,4). Previously, some DES trials investigating the safety and effectiveness of DESs in complex patients with off-label indications showed less favorable results compared with patients with simple on-label characteristics (5,6). In contrast, there have been some reports that have shown favorable outcomes regardless of the lesion complexity after a DES implantation (3,7,8). Consequently, the prognostic effect and clinical outcomes in patients with complex coronary artery disease who have been treated with DESs are debatable. The aim of the present study was to investigate, with the use of a multicenter prospective ZES registry, the differences in the clinical outcomes in patients treated with ZES according to the lesion complexity.

## Methods

### Study design and population

**Figure 1:**
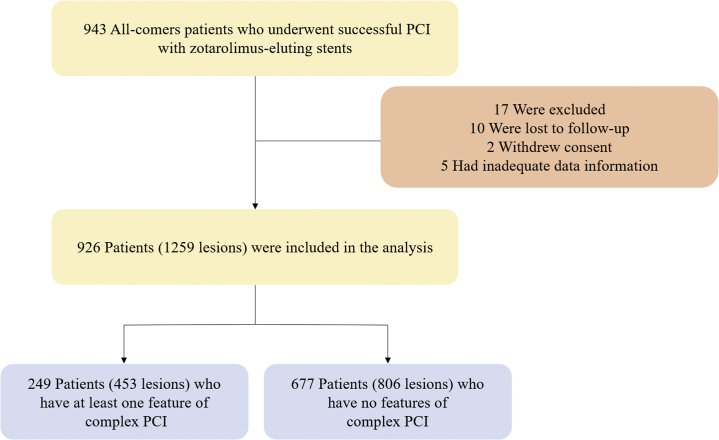
Flow Chart of the Study From the CONSTANT Registry Of a total of 943 patients who underwent a successful percutaneous coronary intervention (PCI) with zotarolimus-eluting stents from the CONSTANT registry, 17 were excluded due to loss to follow-up, withdrawal of consent, or inadequate data, and the resulting 926 patients (1,259 lesions) were divided into 2 groups according to lesion complexity.

The study population was pooled from multicenter, all-comer, observational studies of patients undergoing PCI with second-generation DES from the CONSTANT (Clinical Outcomes iN patientS with zoTArolimus-eluting stent implaNTation) registry. The inclusion criteria, exclusion criteria, and key features of each registry are summarized in Supplemental Table 1. Cardiogenic shock, malignant disease, and patients who had contraindications for aspirin, clopidogrel, and heparin were excluded, and those who received a successful PCI during the index procedure were included. Among the 943 patients who were initially enrolled, 17 were excluded and 926 patients (1,259 lesions) were investigated (Figure 1).

A complex PCI was defined based on a previous report (9,10) as the population who had complex anatomic factors, particularly the presence of at least 1 of the following characteristics: 1) ≥3 lesions were treated; 2) 3 vessels were treated; 3) severe calcified lesions; 4) bifurcated lesions with 2 stents implanted; 5) left main disease; 6) chronic total occlusion lesions; and 7) diffuse long (total stent length ≥60 mm) lesions. They were compared with the noncomplex PCI group. The study protocol was approved by the Keimyung University Dongsan Hospital Ethics Committee (2011-11-512) and the institutional review boards at each participating center, and all patients provided written informed consent for participation in this prospective registry.

### PCI procedures and clinical follow-up

In the CONSTANT registry, balloon angioplasty and stent implantations were performed according to standard techniques. Procedural anticoagulation was achieved with the use of unfractionated heparin. All patients undergoing PCI received a loading dose of dual antiplatelet agents before or during the PCI. After the procedure, dual antiplatelet agents were recommended to be prescribed for a minimum of 12 months after the index procedure. Drugs for secondary prevention were prescribed according to current guidelines.

Clinical follow-up was conducted during hospitalization and after 1, 12, and 24 months, and follow-up coronary angiography was performed at 9 months according to the clinician’s individual discretion. At each visit, information pertaining to the patients’ clinical status, all interventions, and outcome events were recorded. The baseline characteristics and outcome data were collected with the use of a dedicated, electronic case report form by specialized personnel at each participating center. The internet-based system provided each center with immediate and continuous feedback on the processes and quality-of-care measures. Monitoring and verification of the registry data were periodically performed at the participating hospitals by members of the academic coordinating center (Clinical Research Center, Keimyung University Dongsan Hospital, Daegu, Korea).

### Study outcomes and definitions

The primary clinical outcomes were target lesion failures (TLFs), composite end points of cardiac death, target-lesion myocardial infarction (MI), and clinically driven (ischemic symptoms, electrocardiographic changes at rest, or positive stress test) target lesion revascularizations (TLRs). Secondary clinical outcomes included major adverse cardiac events (MACE), the composite end points of death from any cause, MI, any repeated revascularization, and a definite or probable stent thrombosis. Death was considered to have a cardiac cause unless an unequivocal noncardiac cause could be established. The protocol definition of an MI was prespecified and was based on the universal definition of an MI (11): any increase in the cardiac enzymes above the upper range limit with or without the development of Q waves on the electrocardiogram. Periprocedural MI was excluded from the analysis. Repeated revascularization included any type of percutaneous or surgical revascularization procedure and was categorized as a revascularization of any lesion, the target lesion, or the target vessel. A definite or probable stent thrombosis was assessed according to the Academic Research Consortium definition (12). All outcomes of interest were confirmed by the source documentation collected at each hospital and were carefully verified and adjudicated by independent clinicians at each hospital.

### Statistical analysis

Based on the lesion complexity, the data were expressed as n (%) for categoric variables and as mean ± SD for continuous variables. Categorical variables were assessed by means of chi-square tests and Fisher exact tests, and continuous variables were assessed by means of Student *t* tests. Event rates at 2 years and survival curves were generated by means of the Kaplan-Meier method and compared by means of the log-rank test. Unadjusted and adjusted Cox proportional hazard models were used to compare the clinical events according to lesion complexity with an adjustment of the important prognostic covariates such as age, sex, current smoker, history of diabetes, and acute coronary syndrome. In addition, a multivariate Cox regression analysis was used to identify the factors affecting the occurrence of a TLF following the implantation of a ZES, and factors with *P* values <0.10 in the univariate analysis were entered into the multivariate model.

All reported *P* values were 2-sided and were not adjusted for multiple testing. A *P* value <0.05 was considered to be statistically significant. All analyses were performed with the use of SPSS software version 26.0 software and the R programming language.

## Results

### Characteristics of the study patients

From July 2011 to December 2013, a total of 926 patients who underwent a PCI with a ZES were included from the CONSTANT registry. Table 1 presents the baseline demographics and clinical characteristics of the study population according to lesion complexity. The mean age of the patients was 63.83 ± 11.25 years, and about 70% were male. A total of 514 (55.5%) had hypertension and 294 (31.7%) diabetes. The complex PCI group had higher rates of chronic kidney disease (CKD) and dyslipidemia and had a lower ejection fraction than the noncomplex PCI group. Imaging-guided PCI, mainly with the use of intravascular ultrasound (IVUS) but also optical coherence tomography (OCT), was performed in 43.3% of patients. Table 2 presents the lesion and procedural characteristics of the study population according to lesion complexity. A total of 1,259 lesions were treated with ZES. The mean diameter of the stent was 3.06 ± 0.42 mm and the mean stent length was 27.50 ± 13.63 mm. The number of stents was greater, the stent length longer, and the stent diameter larger in the complex PCI group.

**Table 1:** Baseline Demographic and Clinical Characteristics of the Patients

	All (N = 926)	Complex PCI^a^ (N = 249)	Noncomplex PCI (n = 677)	*P* Value
Age, y	63.83 ± 11.25	64.04 ± 10.99	63.75 ± 11.35	0.724
Men	673 (72.7)	191 (76.7)	482 (71.2)	0.095
Body mass index, kg/m^2^	24.42 ± 3.22	24.23 ± 2.94	24.49 ± 3.32	0.280
Diabetes	294 (31.7)	82 (32.9)	212 (31.3)	0.634
Use of insulin	30 (3.2)	8 (3.2)	22 (3.2)	1.000
Hypertension	514 (55.5)	144 (57.8)	370 (54.7)	0.412
Current smoker	324 (35.0)	88 (35.3)	236 (34.9)	0.938
Hyperlipidemia	193 (20.8)	63 (25.3)	130 (19.2)	0.045
Atrial fibrillation	19 (2.1)	6 (2.4)	13 (1.9)	0.608
Previous MI	20 (2.2)	4 (1.6)	16 (2.4)	0.615
Previous PCI	81 (8.7)	16 (6.4)	65 (9.6)	0.149
Previous CABG	13 (1.4)	6 (2.4)	7 (1.0)	0.123
Stroke	90 (9.7)	28 (11.2)	62 (9.2)	0.381
CKD stage ≥4	37 (4.0)	19 (7.6)	18 (2.7)	0.002
Dialysis	26 (2.8)	14 (5.6)	12 (1.8)	0.003
Clinical presentation				0.690
Stable angina	290 (31.3)	75 (30.1)	215 (31.8)	
UA	239 (25.8)	74 (29.7)	165 (24.4)	
NSTEMI	197 (21.3)	62 (24.9)	135 (19.9)	
STEMI	200 (21.6)	38 (15.3)	162 (23.9)	
Multivessel disease	210 (22.7)	115 (46.2)	95 (14.0)	<0.001
Ejection fraction	56.63 ± 11.69	55.51 ± 12.14	57.04 ± 11.50	0.082
Aspirin	920 (99.4)	247 (99.2)	673 (99.4)	0.663
Clopidogrel	809 (87.4)	221 (88.8)	588 (86.9)	0.504
Beta-blocker	680 (73.4)	184 (73.9)	496 (73.3)	0.867
ACEi/ARB	586 (63.3)	146 (58.6)	440 (65.0)	0.078
Statin	817 (88.2)	224 (90.0)	593 (87.6)	0.359
No. of stents per patient	1.57 ± 0.84	2.34 ± 1.10	1.29 ± 0.47	<0.001
Use of IVUS/OCT	401 (43.3)	110 (44.1)	291 (42.9)	0.568
Total procedural time, min	47.22 ± 32.08	62.05 ± 35.58	41.76 ± 28.87	<0.001
Amount of contrast medium, cc	203.63 ± 101.95	251.60 ± 109.50	185.96 ± 93.07	<0.001

Values are mean ± SD or n (%).

ACEi = angiotensin-converting enzyme inhibitor; ACS = acute coronary syndrome; ARB = angiotensin receptor blocker; CABG = coronary artery bypass graft; CKD = chronic kidney disease; IVUS = intravascular ultrasound; MI = myocardial infarction; OCT = optical coherence tomography; PCI = percutaneous coronary intervention; STEMI = ST-segment elevation myocardial infarction; NSTEMI = non–ST-segment elevation myocardial infarction; UA = unstable angina.

a At least 1 of the following characteristics: 3 vessels treated, ≥3 lesions treated, total stent length ≥60 mm, bifurcated lesion with 2 stents implanted, severely calcified lesion, left main disease, and chronic total occlusion lesion.

**Table 2:** Baseline Lesion and Procedural Characteristics

	All (N = 1,259)	Complex PCI (N = 453)	Noncomplex PCI (n = 806)	*P* Value
Target lesion				<0.001
Left main	62 (4.9)	62 (13.7)	0 (0)	
LAD	557 (44.2)	159 (35.1)	398 (49.4)	
LCX	254 (40.2)	88 (19.4)	166 (20.6)	
RCA	386 (30.7)	144 (31.8)	242 (30.0)	
ACC/AHA lesion type				<0.001
A	28 (2.2)	4 (0.9)	24 (3.0)	
B1	280 (22.2)	57 (12.6)	223 (27.7)	
B2	293 (23.3)	91 (20.1)	202 (25.1)	
C	658 (52.3)	301 (66.4)	357 (44.3)	
In-stent restenosis	27 (2.1)	10 (2.2)	17 (2.1)	0.908
Severe CAC	67 (5.3)	67 (14.8)	0 (0)	<0.001
Any bifurcated lesion	258 (20.5)	124 (27.4)	134 (16.6)	<0.001
Bifurcated lesion with 2 stents implanted	36 (2.9)	36 (13.6)	0 (0)	<0.001
Ostial lesion	75 (6.0)	45 (9.9)	30 (3.7)	<0.001
Thrombus present	155 (12.3)	61 (13.5)	94 (11.7)	0.350
Chronic total occlusion	81 (6.4)	81 (17.9)	0 (0)	<0.001
Lesion length, mm	23.62 ± 12.99	28.35 ± 15.82	20.74 ± 9.86	<0.001
Proximal RVD, mm	3.10 ± 0.48	3.13 ± 0.47	3.08 ± 0.49	0.029
Distal RVD, mm	2.84 ± 0.48	2.85 ± 0.48	2.83 ± 0.48	0.583
MLD, mm	0.47 ± 0.31	0.45 ± 0.31	0.48 ± 0.29	<0.058
Diameter stenosis, %	83.77 ± 10.35	84.44 ± 10.83	83.14 ± 10.10	0.015
After NC balloon	230 (18.3)	117 (25.8)	113 (14.0)	<0.001
No. of stents per lesion	1.16 ± 0.38	1.28 ± 0.49	1.09 ± 0.28	<0.001
Stent diameter, mm	3.06 ± 0.42	3.10 ± 0.40	3.04 ± 0.44	0.020
Total stent length per lesion	27.50 ± 13.63	33.01 ± 17.02	24.40 ± 10.15	<0.001

Values are n (%) or mean ± SD.

ACC = American College of Cardiology; AHA = American Heart Association; CAC = coronary artery calcification; LAD = left anterior descending artery; LCX = left circumflex artery; MLD = minimal lumen diameter; NC = noncompliant; RCA = right coronary artery; RVD = reference vessel diameter; PCI = percutaneous coronary intervention.

### Clinical outcomes according to lesion complexity

**Figure 2:**
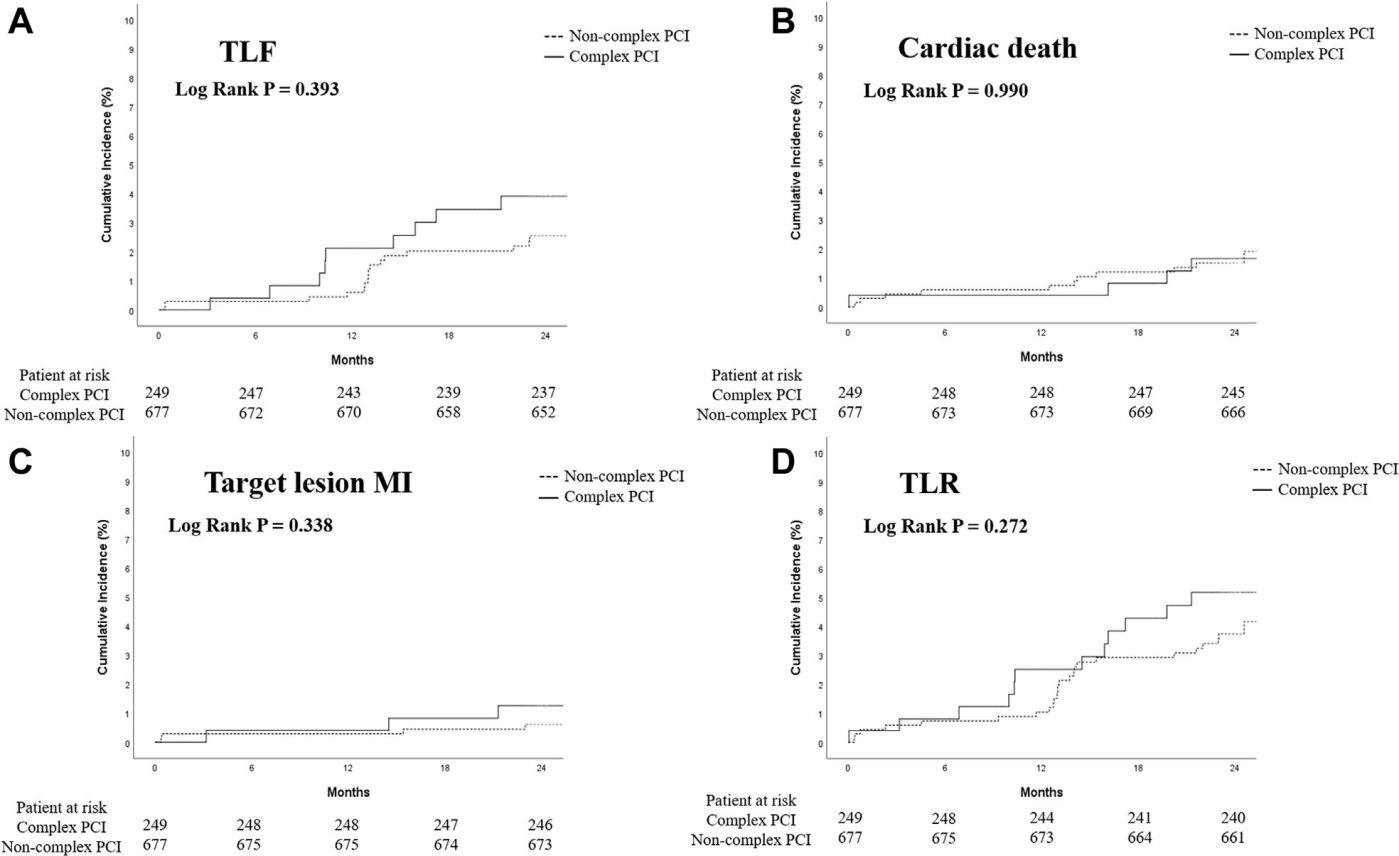
Kaplan-Meier Curve for the 2-Year Clinical Outcomes According to Lesion Complexity Cumulative incidence curves are shown for TLFs stratified by **(A)** lesion complexity, **(B)** cardiac death, **(C)** target-lesion MI, and **(D)** TLR. TLF was defined as a composite of cardiac death, target-lesion MI, and TLR. MI = myocardial infarction; PCI = percutaneous coronary intervention; TLF = target lesion failure; TLR = target lesion revascularization.

At 2 years, the incidence of TLF and its individual components did not differ between the complex and noncomplex PCI groups (4.8% vs 3.7%, respectively; adjusted hazard ratio [aHR]: 1.373, 95% confidence interval [CI]: 0.689-2.738; *P* = 0.367). MACE was also similar (12.0% vs 9.3%; aHR: 1.325; 95% CI: 0.857-2.048; *P* = 0.205) as well as its individual components (Table 3, Central Illustration). The Kaplan-Meier curves of TLF and its individual components are shown in Figure 2. A definite or probable stent thrombosis occurred in 6 patients (0.6%): 3 (1.2%) in the complex PCI group and 3 (0.4%) in the noncomplex PCI group (aHR: 2.819; 95% CI: 0.5-14.025; *P* = 0.205). Table 4 presents the univariate and multivariate Cox regression analyses of the predictors for TLFs. Age ≥70 years and CKD stage ≥4 were independent predictors for TLF within 2 years. In addition, a univariate Cox proportional hazard analysis for TLF according to the number of complex PCI components (Supplemental Figure 1) showed that patients with 3 or more complex PCI components had a statistically and significantly higher TLF rate than the noncomplex group.

**Table 3:** Primary and Secondary End Points According to Lesion Complexity

	All (n = 926)	Complex PCI (n = 249)	Noncomplex PCI (n = 677)	*P* Value	Unadjusted			Multivariable Adjusted^a^
					HR (95% CI)	*P* Value	aHR (95% CI)	*P* Value
TLF	37 (4.0)	12 (4.8)	25 (3.7)	0.451	1.349 (0.678-2.684)	0.394	1.373 (0.689-2.738)	0.367
Death from any cause	34 (3.7)	9 (3.6)	25 (3.7)	0.955	0.982 (0.458-2.104)	0.963	0.971 (0.453-2.083)	0.940
Cardiac	15 (1.6)	4 (1.6)	11 (1.6)	0.984	0.993 (0.216-3.118)	0.990	1.016 (0.322-3.210)	0.978
Noncardiac	19 (2.1)	5 (2.0)	14 (2.1)	0.955	0.974 (0.351-2.703)	0.959	0.917 (0.330-2.550)	0.869
Myocardial infarction^b^	13 (1.4)	5 (2.0)	8 (1.2)	0.352	1.714 (0.561-5.239)	0.345	1.732 (0.560-5.356)	0.340
Target lesion	7 (0.8)	3 (1.2)	4 (0.6)	0.394	2.046 (0.458-9.142)	0.349	2.257 (0.497-10.246)	0.291
Repeated revascularization	64 (6.9)	22 (8.8)	42 (6.2)	0.188	1.466 (0.875-2.456)	0.146	1.478 (0.882-2.479)	0.138
Target vessel	33 (3.6)	12 (4.8)	21 (3.1)	0.231	1.597 (0.786-3.245)	0.196	1.638 (0.804-3.336)	0.174
Target lesion	25 (2.7)	9 (3.6)	16 (2.4)	0.359	1.574 (0.695-3.561)	0.276	1.623(0.715-3.683)	0.247
Nontarget vessel	32 (3.5)	10 (4.0)	22 (3.2)	0.548	1.275 (0.604-2.692)	0.525	1.263 (0.597-2.670)	0.541
Stent thrombosis^c^	6 (0.6)	3 (1.2)	3 (0.4)	0.352	2.740 (0.553-13.575)	0.217	2.819 (0.567-14.025)	0.205
MACE	9 (10.0)	30 (12.0)	63 (9.3)	0.219	1.331 (0.861-2.055)	0.198	1.325 (0.857-2.048)	0.205

Values are n (%) unless otherwise indicated. HRs are for complex PCI compared with noncomplex PCI.

aHR = adjusted hazard ratio; CI = confidence interval; HR = hazard ratio; MACE = major adverse cardiac event; TLF = target lesion failure; PCI = percutaneous coronary intervention.

a Adjusted for age, sex, current smoker, history of diabetes, and acute coronary syndrome.

b Excluded periprocedural myocardial infarctions.

c Included definite or probable stent thrombosis.

**Central Illustration:**
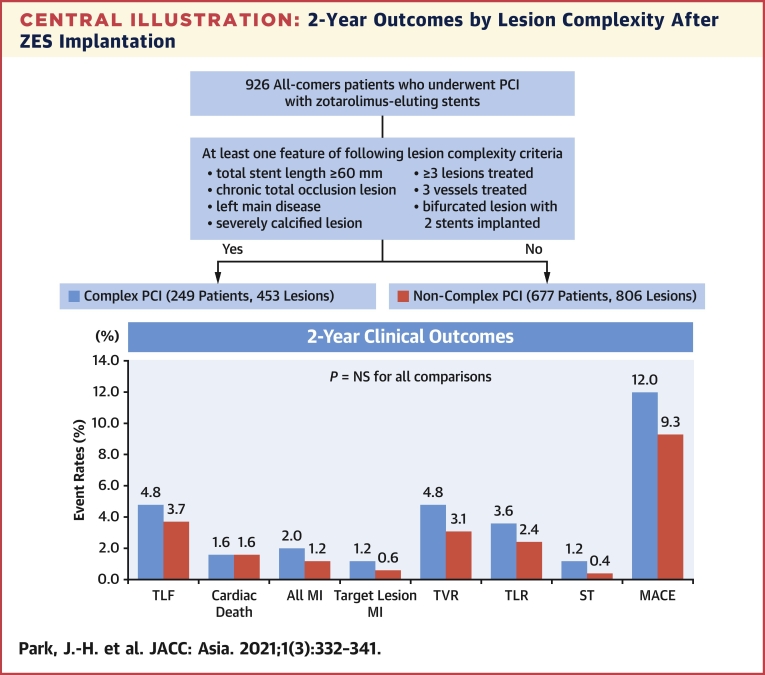
2-Year Outcomes by Lesion Complexity After ZES Implantation A total of 926 patients treated with ZES were divided into complex-PCI and noncomplex PCI groups according to lesion complexity. There were no statistically significant differences of clinical outcomes between the 2 groups over 2 years. MACE = major adverse cardiac event; MI = myocardial infarction; PCI = percutaneous coronary intervention; ST = stent thrombosis; TLF = target lesion failure; TLR = target lesion revascularization; TVR = target vessel revascularization; ZES = zotarolimus-eluting stent.

**Table 4:** Univariate and Multivariate Cox Proportional Hazard Analysis for Predictors of Target Lesion Failure

	Univariate Analysis		Multivariate Analysis^a^	
	HR (95% CI)	*P* Value	aHR (95% CI)	*P* Value
Age ≥70 y	2.117 (1.057-4.238)	0.034	2.830 (1.460-5.487)	0.002
Acute coronary syndrome	1.351 (0.607-3.008)	0.461		
Diabetes	2.571 (1.284-5.148)	0.008	1.653 (0.854-3.198)	0.136
CKD stage ≥4	5.259 (2.100-14.190)	<0.001	3.433 (1.157-10.185)	0.026
LVEF <40%	1.628 (0.627-4.228)	0.317		
Complex PCI	1.349 (0.678-2.684)	0.394	1.215 (0.602-2.450)	0.587
Bifurcated lesion with 2 stents	3.007 (0.723-12.503)	0.130		
Total stent length ≥60 mm	2.657 (1.313-5.378)	0.007		
Use of IVUS/OCT	0.705 (0.359-1.385)	0.310		

aHR = adjusted hazard ratio; CI = confidence interval; CKD = chronic kidney disease; HR = hazard ratio; IVUS = intravascular ultrasound; LVEF = left ventricular ejection fraction; OCT = optical coherence tomography; PCI = percutaneous coronary intervention.

a A multivariable cox regression analysis was performed by including the variables with a *P* value of <0.10, except for a total stent length of ≥60 mm due to the variable being a component of a complex PCI. Variables included in the multivariate analysis model: Age ≥70 years, diabetes, CKD ≥ stage 4, and a complex PCI.

## Discussion

The main findings of the analyses of the impact of the lesion complexity on the clinical outcomes in patients treated with ZES were: 1) In an all-comer cohort, the complex PCI population represented ∼27%; 2) ZES had a similar mid-term outcome in the complex PCI group as in the noncomplex PCI group; and 3) older age (≥70 years) and advanced CKD (stage ≥4) were prognostic factors for TLFs over 2 years.

**Figure 3:**
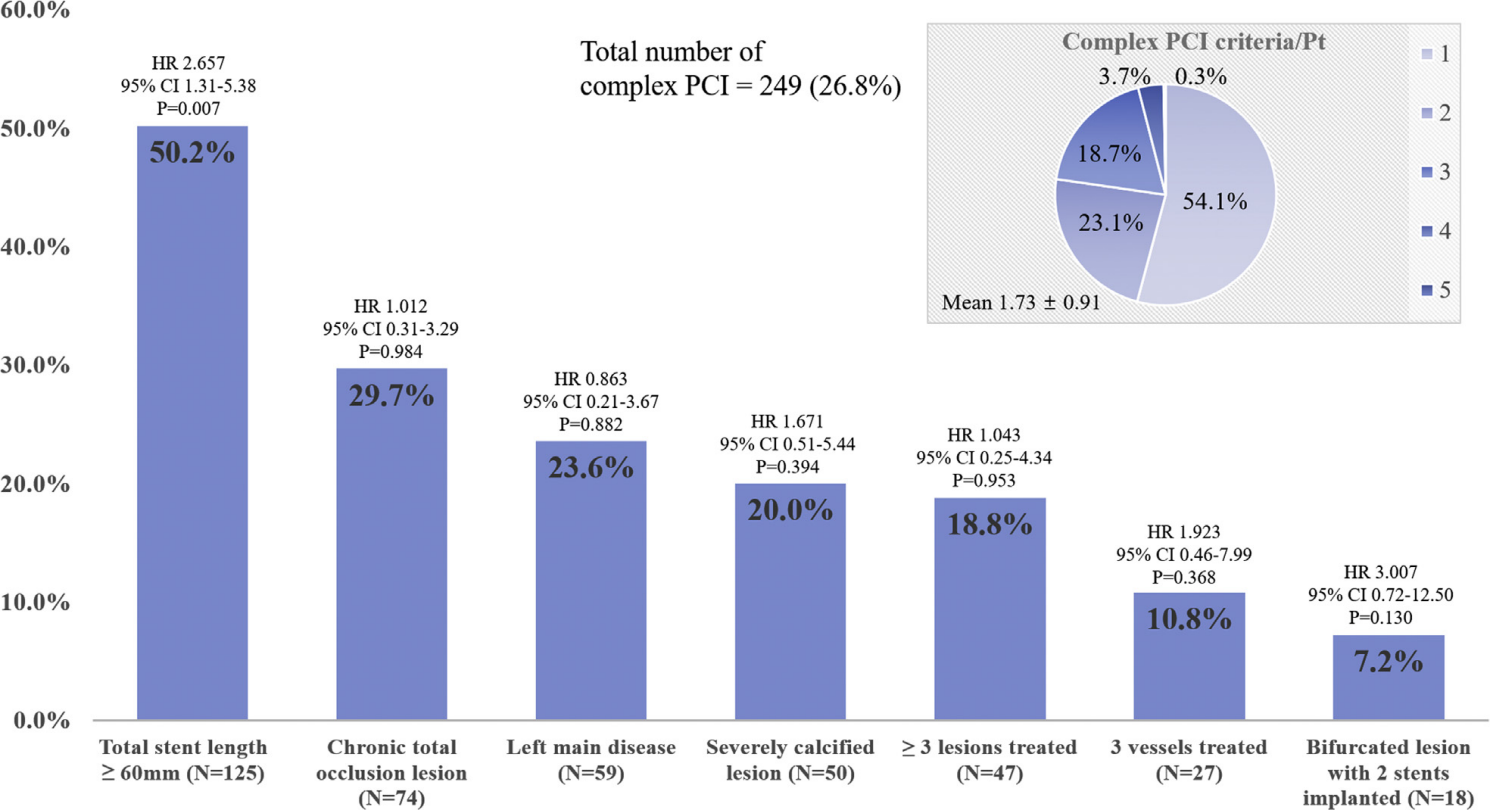
Prevalence of the Individual Qualifying Variables in the Complex PCI Group The definition of complex PCI required fulfillment of at least 1 of the following: 1) ≥ 3 lesions treated; 2) 3 vessels treated; 3) severe calcified lesions; 4) bifurcated lesions with 2 stents implanted; 5) left main disease; 6) chronic total occlusion lesions; and 7) diffuse long (total stent length ≥60 mm) lesion. This figure shows the distribution of the qualifying characteristics within the complex PCI group (n = 249; 26.8%). The HR and 95% CI for TLF of each component was recorded. CI = confidence interval; HR = hazard ratio; PCI = percutaneous coronary intervention = TLF = target lesion failure.

There is no universally agreed definition of what constitutes lesion complexity (2,9,10), and the definition of a complex PCI in this study was based on the complex PCI groups and subgroups in recent trials (7,10,13), which have at least 1 of the following characteristics: 1) ≥ 3 lesions treated; 2) 3 vessels treated; 3) severe calcified lesions; 4) bifurcated lesions with 2 stents implanted; 5) left main disease; 6) chronic total occlusion lesions; and 7) diffuse long (total stent length ≥60 mm) lesions. The prevalence of each component of the complex PCI definition and HRs for TLF with 95% CIs is shown in Figure 3. Among the patients in the complex PCI group, 45.9% met at least 2 criteria, and a total stent length of ≥60 mm was the most common characteristic (50.2%).

In the DES era, PCIs for the high-risk group, whether they involved lesion complexity or clinical complexity, have been emerging and increased the size of this cohort. There is a degree of consensus on expected poor prognosis in patients with clinical complexity (14,15). Similarly, a high-risk clinical background such as an old age, CKD, diabetes, and poor hemodynamics is still an important risk factor (9,13), and it could be understood that the SYNTAX II score, which is a clinical tool that combines the clinical variables with the anatomic SYNTAX I score, provides a safer and more effective treatment strategy between PCI and coronary artery bypass grafting (15,16). However, the prognostic effects of a PCI in patients with lesion complexity have some uncertainty according to earlier studies. Consequently, the present study focused on the lesion complexity itself with the use of biocompatible stents, and not on patient complexity.

Mohamed et al (5) showed that patients with complex target lesions are at an increased risk of poor clinical outcomes compared with noncomplex PCI patients (TLF: 4.2% vs 2.8%; *P* < 0.0001), and a greater number of complex PCI features correlates with a higher mortality and worse outcomes. Généreux et al (2) reported similar adverse outcomes in a complex PCI group over 2 years (MACE: 9.7% vs 5.3%; *P* < 0.0001). On the other hand, there are some reports that have shown similar outcomes in the complex PCI group. Endo et al reported the clinical impact of a complex PCI with a second-generation DES (13). The occurrence of death from any cause tended to be higher in the complex PCI group than in the noncomplex PCI group, but it was not significant (4.5% vs 2.6%; *P* = 0.10); the same trend was present for major adverse cardiac and cerebrovascular events (composite of cardiac or cerebrovascular death, nonfatal MI, and nonfatal ischemic strokes; 2.5% vs 2.0%; *P* = 0.58). Furthermore, the Onyx One Clear (A Single Arm Trial With Resolute Onyx in One-Month DAPT for High-Bleeding Risk Patients Who Are Considered One-Month Clear; NCT03647475) complex PCI subgroup analysis showed the safety and effectiveness of a 1-month short dual antiplatelet therapy among patients at high bleeding risk treated with ZESs regardless of lesion complexity (cardiac death/MI: 9.3% vs 6.1% [*P* = 0.46]; TLR: 4.5% vs 2.9% [*P* = 0.67]) (7). In the TWILIGHT (Ticagrelor With Aspirin or Alone in High-Risk Patients After Coronary Intervention; NCT02270242) substudy, there was no significant statistical interaction of the treatment effects on the ischemic end points between the complex and noncomplex PCI groups up to 1 year (10). Kang et al (17) showed that the clinical factors seem to have a greater and more prolonged effect on the outcomes than the procedural factors. As such, there is some debate on the clinical impact of lesion complexity in the real-world population. A comparison table of the DES clinical trials in complex PCIs is presented in Supplemental Table 2.

The results in the complex PCI group in the present study have shown comparable clinical outcomes to the noncomplex PCI group (TLF: 4.8% vs 3.7%; aHR: 1.373; 95% CI: 0.682-2.738; *P* = 0.367; MACE: 12.0% vs 9.3%; aHR: 1.325; 95% CI: 0.857-2.038; *P* = 0.205) even after an adjustment with multivariable analysis. There are several possible explanations for these results: first, the development of a stent platform that enhances deliverability and flexibility, especially in complex lesions; second, a high proportion of an extended dual antiplatelet therapy (beyond 1 year) in the complex PCI group (85.7% vs 80.7%; *P* = 0.097), and third, the relatively higher percentage of intravascular imaging use (IVUS/OCT, 43.3%) with stent optimization (18,19). In multivariate Cox proportional hazard analysis for the predictors of TLF, patient factors such as CKD stage ≥4 and age >70 years were found to be predictors of TLF, but overall lesion complexity did not affect the clinical outcomes. It is reasonable that the favorable outcomes in the complex PCI group in this study might have affected the definition of a complex PCI, which did not include the important clinical covariates. Nevertheless, this result might help to decide the revascularization strategy in patients with complex coronary artery disease, because there is no definite consensus on the impact of lesion complexity, as mentioned above. In conclusion, with the development of biocompatible stents and improvement in PCI techniques, the results of this study suggest that PCI for a complex lesion with the use of a ZES might be comparatively safe for up to 2 years. Further large-cohort studies on the impact of concomitant high-risk lesion complexity are needed.

### Study limitations

First, it was an observational nonrandomized trial and the 2 classified groups were retrospectively enrolled. Consequently, the overall findings must be considered as hypothesis generating only. Second, because the data were from observational registries, the clinical events may not have been carefully taken, and the patient follow-up might not have been as strict as it would have been in a randomized trial. This could be the reason for the lower event rates in this study, predominantly with the rate of target-lesion MIs, which was much lower in our study than in an earlier randomized controlled trial. However, the relatively higher incidence of an image-guided PCI (43.3%) with optimization of the stent implantation might be one of the reasons for the low event rate. Data from the National Inpatient Sample from 2007 to 2013 in the United States showed that the IVUS use rate was 6.9% in 2007 and 8.8% in 2013 (19), and the results of the European Association of Percutaneous Cardiovascular Interventions showed that 10.4% of coronary intervention cases were guided by intravascular imaging (18). Third, there might have been hidden confounding factors, such as individual patient complexity, even if the data were adjusted by multivariate analysis with acute coronary syndrome (ACS) and there were no statistical differences in the proportion of the ACS component between the 2 groups. However, we defined the complex PCI composite without consideration of the differences between the individual impacts of patient factors such as ACS and its importance on the clinical outcomes. Fourth, the evaluation of severe calcification might have been underestimated, because at the time of the index procedure, IVUS/OCT was not mandatory and was conducted individually according to the discretion of the physician. Fifth, because the patients who received successful PCI during the index procedure were selected in the inclusion criteria and because complex PCI has a certain incidence of TLR during the procedure, there might have been a chance of an underestimation in the complex PCI population. Finally, owing to the relatively small sample size compared with other large-scale cohort studies, the post hoc power analysis of TLF value was 12.8%. Further longitudinal studies on larger populations are needed to clarify the clinical significance of the clinical and anatomic factors. Despite these limitations, our data showed similar clinical outcomes in the complex and noncomplex PCI groups.

## Conclusions

In the second-generation DES era, especially with ZESs, the clinical end points of TLF and MACE did not significantly differ according to lesion complexity for up to 2 years.

Perspectives **COMPETENCY IN MEDICAL KNOWLEDGE:** With the development of second-generation DESs, they are frequently being used to treat complex coronary lesions. However, there are conflicting data regarding the clinical outcomes according to lesion complexity after DES implantations. This study investigated the clinical outcomes of complex versus noncomplex PCIs after ZES implantations in all-comer Korean patients. The 2-year TLF rate was similar between the 2 groups, and independent predictors of 2-year TLF were older age and advanced chronic kidney disease. The DES system, including a cobalt alloy stent platform and biocompatible polymers, and optimal medical treatment may have beneficial effects in complex PCI.

**TRANSLATIONAL OUTLOOK:** This study was an observational registry and reported the mid-term clinical outcomes according to lesion complexity after ZES implantations. Therefore, a randomized trial with a longer-term follow-up of complex PCIs is needed to clarify the impact of lesion complexity on clinical outcomes for the current generation of DESs.

## Funding Support and Author Disclosures

Dr Hur has received research grants from Medtronic. All other authors have reported that they have no relationships relevant to the contents of this paper to disclose.
