# Supplementary material for: Clinical Impact of Lesion Complexity on 2-Year Outcomes After Zotarolimus-Eluting Stents Implantation
Source: JACC Asia. 2021 Oct 26;1(3):332–41. doi: 10.1016/j.jacasi.2021.08.006 (PMC9627827; doi:10.1016/j.jacasi.2021.08.006)
Supplement: Supplemental Tables 1 and 2 and Supplemental Figure 1 [file mmc1.docx]

**Supplemental Table 1. Key Features of CONSTANT Registry**

| Design | CONSTANT |
| --- | --- |
| Study type | \| 7 Centers, prospective, nonrandomized observational registry including PCI with R-ZES \| \| --- \| |
| Stent name | \| Resolute Integrit \| \| --- \| |
| Stent type used for PCI | \| Zotarolimus-eluting stents \| \| --- \| |
| Main inclusion criteria | \| DS for epicardial coronary artery ≥70%, or 50%–70% with objective evidence of myocardial ischemia; diameter stenosis for left main coronary artery ≥50%, and amenable to PCI. \| \| --- \| |
| Exclusion criteria | \| The exclusion criteria were minimal. Patients with cardiogenic shock, malignant disease, or other comorbid conditions with a life expectancy of <12 months; those treated with a mixture of different types of DES; and those with planned surgery necessitating interruption of antiplatelet drugs within 6 months after the procedure were excluded. \| \| --- \| |
| Recruitment period | Jul 2011 – Dec 2013 |
| Follow-up period, years  (median and IQR) | 2.02 (1.97-2.07) |

Abbreviations: DES, drug-eluting stent(s); DS, diameter of stenosis; IQR, interquartile range; PCI, percutaneous coronary intervention; R-ZES, Resolute zotarolimus-eluting stent

**Supplemental Table 2. Comparison of the Cinical Trials of DESs in Complex PCIs**

| **Title of trial**  **or author** | **Number of**  **complex-PCI** | **Number of**  **noncomplex PCI** | **DES** | **Components of**  **complex PCI** | **Follow-up**  **duration** | **Clinical outcomes** |
| --- | --- | --- | --- | --- | --- | --- |
| TWILIGHT | 2342 | 4777 | ? | Lesion complexity only | 1 year | Cardiac death 1.2% vs. 0.8%, p=0.39  Myocardial infarction 3.2% vs. 2.5%, p=0.32  Stent thrombosis 0.6% vs. 0.4%, p=0.52 |
| Endo et al. | 358 | 704 | ? | Lesion complexity only | 3 years | TVF 12.0% vs. 6.8%, p<0.01  Cardiac death 1.7% vs. 1.1%, p=0.18  Non-fetal MI 0% vs. 0.1%, p=0.36 |
| Onyx One Clear | 401 | 1105 | R-ZES | Lesion complexity only | 1 to 12  months | TLF 10.7% vs. 7.3%, p=0.99  Cardiac death 2.8% vs. 2.6%, p=0.92 |
| Substudy of  RESOLUTE  all-comers | 1602 | 772 | ZES/EES | Lesion complexity  + Clinical covariates | 1 year | TLF 9.3% vs. 6.3%, p=0.01  POCO 16.1% vs. 11.6%, p<0.01 |
| Genereux et.al. | 2255 | 6327 | Mainly  EES | Lesion complexity only | 2 years | MACE 9.7% vs. 5.3%, p<0.01  Death 4.9% vs. 3.5%, p<0.01 |
| Mohamed et al. | 9793 | 25596 | SES | Lesion complexity only | 1 year | TLF 4.2% vs. 2.8%, p<0.01  POCO 7.9% vs. 6.0%, p<0.01 |
| Win et al. | 1817 | 1506 | SES/PES | Lesion complexity  + Clinical covariates | 1 year | MACE 17.5% vs. 8.9%, p<0.01  Stent thrombosis 1.6% vs. 0.9%, p=0.05 |
| CONSTANT  registry | 249 | 677 | R-ZES | Lesion complexity only | 2 year | TLF 4.8% vs. 3.7%, p=0.75  Stent thrombosis 1.2% vs. 0.4%, p=0.18 |

Abbreviations: EES, everolimus-eluting stent; MACE, major adverse cardiac event; MI, myocardial infarction; PES, paclitaxel-eluting stent; POCO, patient oriented clinical outcome; PCI, percutaneous coronary intervention; SES, sirolimus-eluting stent; TLF, target lesion failure; TVF, target vessel failure; ZES, zotarolimus-eluting stent

**Supplemental Figure 1. TLF According to the Number of Complex PCI Component**


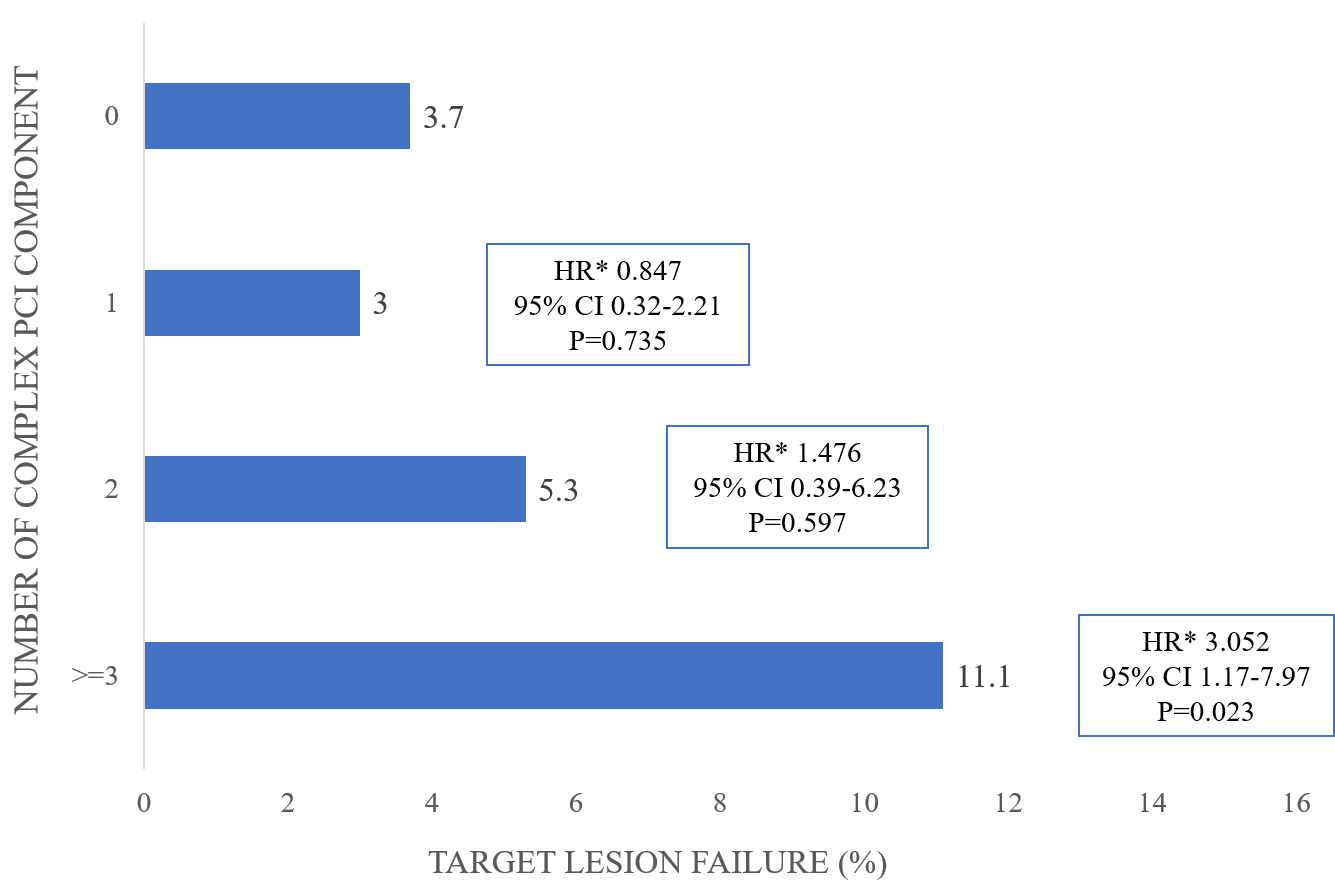


A univariate cox proportional hazard analysis for a TLF according to the number of complex PCI component showed that in patients with 3 or more complex PCI components had a statistically significantly higher TLF rate than non-complex group.

*Hazard ratios were for each group (1 or 2 or ≥3 of complex PCI components) compared to a non-complex PCI group.

Abbreviations: HR, hazard ratio; CI, confidence interval; PCI, percutaneous coronary intervention; TLF, target lesion failure
